# Teaching and learning pharmacology in Brazil before COVID-19 pandemic: a case study in Rio de Janeiro

**DOI:** 10.1186/s12909-023-04437-4

**Published:** 2023-06-23

**Authors:** Antonio Augusto Fidalgo-Neto, Renato Matos Lopes, Ricardo Riedel Martins Ribeiro, Cristina Alves Magalhães de Souza, Raul Luiz de Souza Cavalcanti, Natiele Carla da Silva Ferreira, Luiz Anastacio Alves

**Affiliations:** Laboratory of Cellular Communication, Oswaldo Cruz Foundation, Av. Brasil, 4365, Manguinhos, Rio de Janeiro, Brazil

**Keywords:** Pharmacology, Medical schools, Medical education, Brazil, Pharmacology curricula

## Abstract

**Background:**

Knowledge of pharmacology is crucial for physicians to perform rational and safe medicine. Medical professionals are responsible for prescribing drugs and a weak performace of those can result in medication errors leading to disability, hospitalization, and death, among other situations. It occurs worldwide, including in Brazil, so that learning pharmacology impacts on public health service. We aim to investigate the current pharmacology educational practices in medical schools in the state of Rio de Janeiro, Brazil.

**Methods:**

We surveyed 14 of 22 medical schools in Rio de Janeiro. Pharmacology teachers (*n*=16) and medical students (*n*=89) answered a semi-structured questionnaire that included questions about the staff characteristics, pharmacology content, teacher’s concepts, and common practices and resources that were used in pharmacology classes.

**Results:**

Our results revealed that the medical schools had similar overall curriculums. Pharmacology teachers work more than 30hs a week (75%) and conducted both research and teaching (62.5%). We also found that the multimedia projector was the most common resource (71.9%), and passive pedagogical methodologies (e.g., expository classes) remain a current strategy in pharmacology classes (89.9%). In general, medical students are poorly motivated (55%), which may be related to their performance in assessments. In addition, students believe that pharmacology is a complex (52%) or very complex subject (46%) since for its full understanding the student needs concepts from other disciplines, which can have an impact on the performance and motivation of students. As a result, these medical students do not fully understand the integration between pharmacology’s basic concepts and their clinical applications.

**Conclusion:**

These data seem to demonstrate that the adopted teaching and learning pharmacology strategies and methodologies can be improved in Rio de Janeiro.

**Supplementary Information:**

The online version contains supplementary material available at 10.1186/s12909-023-04437-4.

## Background

Medical education should be a vital issue in the globalization process due to the mobilization of the population, physicians and pandemic diseases. In this context, investigations into the disciplines involved in medical education are essential to improving the actual scenario. Brazil is ranked second in the world in terms of the number of medical schools, with approximately 361 and 216 million people in 2023 [1]. The medical course lasts six years and the time required for specialization can be from 2 to 5 years, depending on the type of specialty and area of activity chosen. The majority of them follow a traditional model of teaching, with some using problem-based learning and hybrid systems. A more detailed review of medical education in Brazil was recently published [2, 3].

In this context, pharmacology is a mainstream basic science in the study of Medicine and is one of the crucial subjects found in basic and clinical medical curricula [4-9]. However, teaching and learning pharmacology is a complex task. Medical students are expected to learn a significant amount of information by the time they graduate. As the understanding of pharmacology and pharmacotherapeutics may demands the knowledge of nearly 20,000 therapeutic agents [10], the inclusion of this subject in a medical curriculum significantly increases the quantity of information that students need to learn. The primary objective of learning pharmacology is to motivate medical students to gain knowledge of the general pharmacological and therapeutic principles that aid in the effective management of diseases. Medical students should develop the ability to sort out pharmacological information and concepts from the overload of information that is presented to them, be able to integrate this information into clinically relevant situations‚ and apply this knowledge in the management of a patient’s illness [11, 12]. Additionally, pharmacology is more than a distinct subject in medical education; it is an interdisciplinary subject that integrates basic science (e.g., Biochemistry, Physiology, Toxicology, and Pharmacy) as well as clinical science (e.g., cardiovascular pharmacology, neuropharmacology, psychopharmacology, therapeutic‚ and clinical pharmacology) [11, 12].

Despite requiring a teaching strategy that encourages learning through interdisciplinary logical reasoning, in practice the teaching of pharmacology is still limited to the traditional didactic methodologies (e.g., content-based expositive classes) [10]. In this scenario, some authors have highlighted the need to review the teaching practices in pharmacology education [4, 5, 11-15]. The teaching and learning of the pharmacological sciences within medical curricula require a novel, effective and holistic approach to motivate medical students to learn the essential objectives of this subject. Scientific advances in the field should lead to frequent changes in the curriculum, which would represent a challenge due to its overload [16].

Learning in pharmacology generates impacts on public health since medical professionals will be responsible for prescribing drugs. Prescribing is a complex and challenging task that requires knowledge of pharmaceuticals and an understanding of the principles of clinical pharmacology among other skills [17, 18]. Studies from different countries pointed out that approximately 50 to 80% of medical students or junior doctors were not confident to prescribe or had problems to apply the pharmacology knowledge in their clinical practices [19-21].

A growing number of studies and epidemiological data demonstrate that medication errors are present in different situations of health care. It is important to emphasize that medication errors can cause life-threatening situations, disability, birth defects, hospitalization, and death. The American Food and Drug Administration (FDA) agency reported that they receive about 100,000 notifications annually suspected of medication errors [22]. Medication errors also cause approximately, 7,000 to 9,000 deaths each year only in the United States as well as a high cost (some billions of dollars) to treat patients suffering adverse effects associated with these errors [23].

Despite Brazilian data concerning drug administration errors or adverse effects are scarce and under-registered [24]. Several reports on prescription errors and adverse effects that are caused by drug-drug interactions in Brazilian hospitals have been reported [25-28]. A recent qualitative Brazilian study in a hospital showed that an important error is that the prescriptions of junior doctors are not checked by other healthcare professionals [29]. In keeping with the idea that it is crucial to check prescriptions in a Brazilian teaching hospital, the pharmacists found 11.5% errors in a total of 1.874 prescriptions [30].

A clinical practice that is safe and free of damage is considered a global objective, as presented in the document Global Patient Safety Challenge on Medication Safety of the World Health Organization (WHO). Nevertheless, investments are necessary for the development of systems, practices and technologies that can prevent errors and improve drug therapy [17, 18].

Pharmacology education plays a central role in the safe practice of medicine as medication-induced adverse events, including drug-drug interactions, occur frequently and it can harm public health. Thereby raising major concerns about the adequacy of pharmacology education in medical schools [4, 5, 31, 32]. These reports emphasize the need to improve the ways that current and unbiased pharmacological knowledge is gathered and kept up to date for medical students and medical practitioners. These reports also highlight the need for further investigation of the teaching practices that are used in current pharmacology education.

The present study aims to describe the current pharmacology education practices in the medical schools of the state of Rio de Janeiro, Brazil before the COVID-19 pandemic. The first objective was to identify the main characteristics of the existing medical schools in the state. The second objective was to outline a profile of pharmacology teachers from universities in the same state. Finally, the present study aimed to explore the medical students’ perceptions of pharmacology as a subject within the medical curriculum.

## Methods

### Study subjects

The present study was divided into two parts. The first part focused on the medical schools and the pharmacology teachers. The second part focused on the medical students who finished the pharmacology subject of their program. The survey was carried out between 2009 and 2010; however, the number of medical schools and curricula were updated in 2022.

### Medical schools

We planned this study based on O'Shaughnessy and colleagues [33], who described the teaching of clinical pharmacology and therapeutics in United Kingdom medical schools. According to the Ministry of Education (https://emec.mec.gov.br/), the Brazilian department responsible for accrediting and supervising educational institutions, there are 22 active medical schools in the state of Rio de Janeiro in 2022. However, only 14 schools were included in the present study. One medical school was excluded because it does not include pharmacology in its curriculum, since it adopts a problem-based learning (PBL) curriculum. A further 7 medical schools were not included in the survey since they have less than 10 years of activity.

### Pharmacology teachers

After identifying the number of medical schools in Rio de Janeiro, we searched their websites for organizational and curricular data (including the workload of the pharmacology discipline, the period in which it was offered, and the professors responsible for teaching this discipline). Based on this information, we carried out a new search on the Lattes platform - a database of curricula and institutions in the science and technology areas in Brazil (it is available in Portuguese and English at https://lattes.cnpq.br/) to identify the basic education, the degree level, the workload of these professors and whether they carried out other activities, including research and scientific guidance.

The pharmacology departments of all 14 medical schools were invited to participate. The head of each department, or one of his or her staff, was contacted by phone or e-mail and was asked to answer a questionnaire that was sent to them by e-mail (Supplementary Information). The participants have been working for more than 1 decade as pharmacology teachers.

### Medical students

Eighty-nine medical students from the 14 medical schools in the present study were randomly selected to respond to the in-house questionnaire. All the students had concluded the pharmacology course (which occurred between the third and fifth year in school).

### Questionnaires

In this study, we used two questionnaires, one for pharmacology teachers and the other for medical students. The questionnaires were developed according to the model used by O'Shaughnessy and colleagues [33] and Tables 1 and 2 summarize their target aspects. All questionnaires were tested and validated in small groups of volunteers (around 10% of the total n) before being applied to improve the final version (see Additional files 1, 2, 3 and 4). All volunteers signed the Free Informed Consent.

**Table 1:** Analyzed information from the medical schools and the Pharmacology teachers

**Staff characteristics**
- Teacher’s basic education
- Teacher’s graduate level
- Teacher’s total working time
- Teacher’s other occupation(s)
- Number of pharmacology teachers per medical school
**Pharmacology content and general organization**
- Number of pharmacology-related subjects (ex., basic and clinical pharmacology)
- Pharmacology curriculum
- Textbook adopted
**Teacher’s perceptions, experiences, and ideas**
- Level of difficulty compared with other medical subjects (to teach and learn)
- Knowledge’s dependency on other medical subjects
- Overall students’ performance (approval and abandon)
**Common practices and resources**
- Learning approaches (lectures, seminars, tutorials, and self-directed study, among others)
- Pedagogical orientation
- Whether students are motivated to search for other sources of information (scientific papers and websites, among others)
- Laboratory practical classes (use of animals or no use of animals)
- Availability of textbooks and other references in the medical school library
- Computer use and Internet access provided by medical school
- Pedagogical resources (blackboard, smart board, multimedia projector, and slide projector, among others)
- Use of educational software as a pedagogical tool

**Table 2:** Analyzed information from medical students

**Students’ perceptions concerning pharmacology content, pedagogical preferences, and grades**
- Motivation and satisfaction during pharmacology classes (Likert Scale, 1 – 4)
- During pharmacology classes, do you prefer:
1. A traditional expository lecture. The teacher presents all the pharmacology content. (passive learning)
2. The pharmacology content is presented through problems and tests in a clinical context. The expository lecture is not performed.
3. The pharmacology content is presented through problems and tests in a clinical context and then, the teacher presents the content with an expository lecture.
4. Other (please explain)
- What pharmacology textbook did you use?
- What do you think about the use of computers and educational software as a tool to facilitate teaching and learning in pharmacology?
- Comparative pharmacology’s level of importance with the other medical subjects (Likert Scale, 1 – 4)
- Did you use textbooks of a different subject to study pharmacology? (e.g., biochemistry, physiology, chemistry, and others)
- Final grades (A, B, C, or D)
**Common practices and resources**
- Learning approaches (lectures, seminars, tutorials, and self-directed study, among others)
- Usage of clinical examples during classes (Likert Scale, 1 – 4)
- Pedagogical resources (blackboard, smart board, multimedia projector, and slide projector, among others)
- An interdisciplinary approach to teaching was used (Likert Scale, 1 – 4)
- Textbook adopted
- Laboratory practical classes (use of animals or no use of animals)
- Students’ assessments (written tests, oral tests, seminars, projects, and self-assessments, among others)

The questionnaires were largely composed of closed questions, but they also included several open questions; thus, these questions allowed a qualitative evaluation. When necessary, semi-structured interviews were conducted with the teachers**.** Furthermore, the survey questionnaire contained several questions that required the respondents to reply using a 4-point Likert scale rating. Likert scaling is the most widely used psychometric scale in survey research. It asks respondents to indicate their levels of agreement with a declarative statement [34]. Therefore, Likert scales are used for measuring opinions, psychic and mental dispositions, and preferences [34, 35]. A Likert scale was used to measure their level of satisfaction and motivation concerning the pharmacology lectures. The scale ratings ranged from 1 to 4, according to the following sequence: 1 for unmotivated, 2 for poorly motivated, 3 for motivated, and 4 for very motivated. The 4-point Likert scale was also used to measure the frequency that a certain didactic event occurred (clinical examples, interdisciplinary classes, and need for other knowledge), according to the following sequence: 1 for non-occurrence, 2 for rarely, 3 for eventually, and 4 for frequently.

### Statistical analysis

Data were organized in spreadsheets and quantified (average, median, percentages, minimum, and maximum) using the Microsoft Excel program version 2212 for Microsoft 365 MSO. The graphics were prepared using the same program.

## Results

### Medical schools

**Fig. 1:**
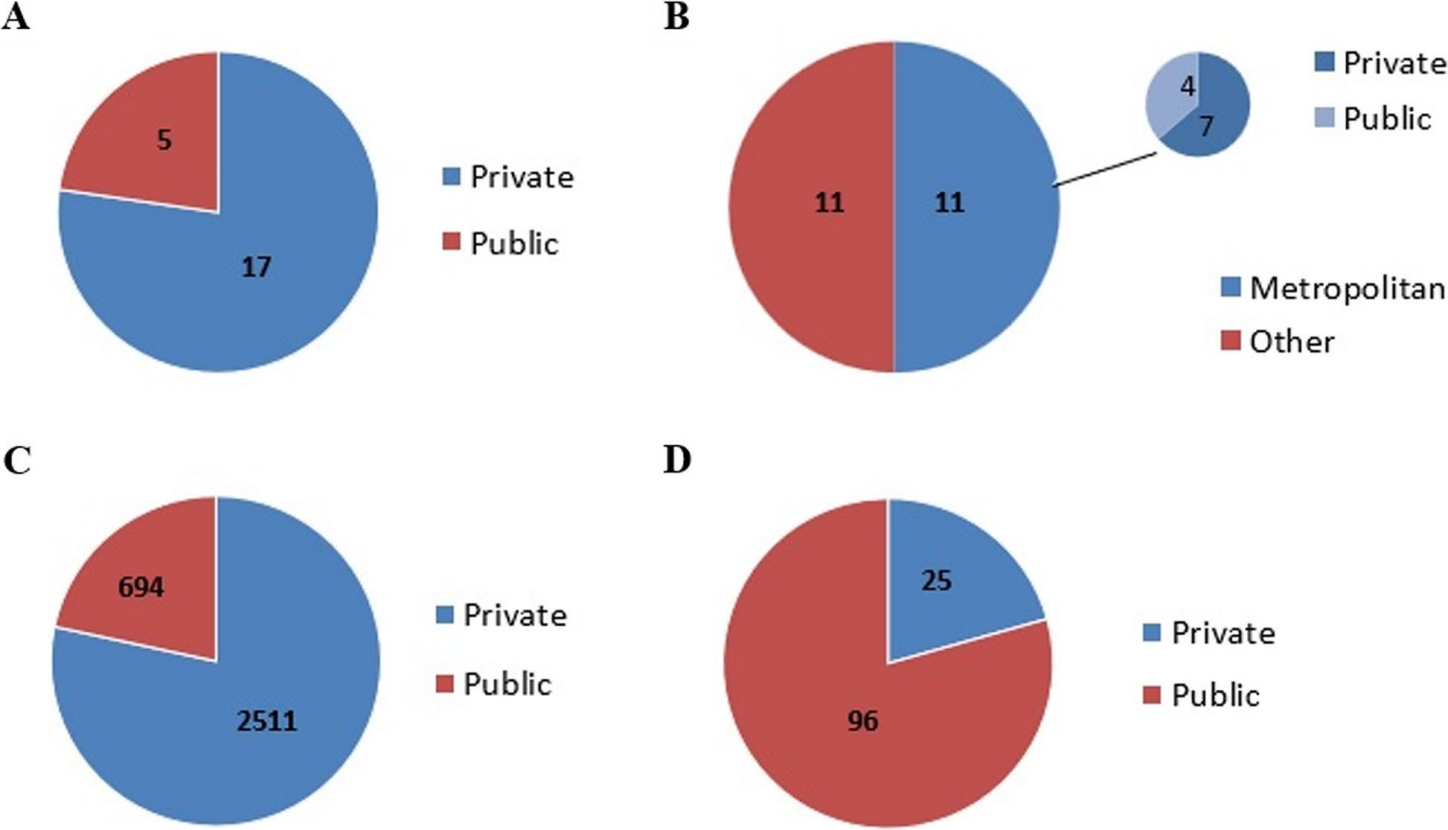
Main characteristics of medical schools in the state of Rio de Janeiro. Administration of the 22 medical schools in activity in the state (**A**). Localization of the 22 medical schools in the state of Rio de Janeiro (**B**). The absolute number of vacancies offered per year by public and private medical schools (**C**). Median activity time (in years) of public and private medical schools in the state of Rio de Janeiro (**D**)

Based on the Ministry of Education platform there are 22 medical courses in activity in the state of Rio de Janeiro, with 17 courses offered by private teaching institutions and 5 courses offered by public institutions, as shown in Fig. 1A. Most medical schools are concentrated in the metropolitan region of the state of Rio de Janeiro, i.e., 11 schools, of which 4 are public and 7 are private as demonstrated in Fig. 1B. The other regions of the state have only private medical schools, except the North region, which has a public medical school.

Private schools provide approximately 78.3% of vacancies in the Medicine course (i.e., 2511 vacancies per year), and public schools offer 694 new vacancies per year as illustrated in Fig. 1C.

Public medical schools in the state of Rio de Janeiro are centenary, with a median time of activity of 96 years, with the youngest school having been in operation for 13 years, while the oldest has been in operation for 214 years. On the other hand, private schools have a median time of activity of 25 years (minimum= 4 years; maximum= 55 years), as shown in Fig. 1D.

The composition of the curricula among the 14 selected medical schools was very similar. Pharmacology content was offered by 12 of the 14 selected universities. It was often divided into two or more subjects such as pharmacology II, applied pharmacology, or clinical pharmacology (*n*= 9) that were offered during different periods (min= second year; max= fourth year). The total credit hours for pharmacology range between 60 to 150 h for basic pharmacology and 60 to 240 h for pharmacology II/applied pharmacology or clinical pharmacology, as shown in Table 3.

**Table 3:** Pharmacology curricula from selected universities

**Subject**	**Total medical schools (*n*= 14)**	**Subject available in the year**	**Workload**
**Basic pharmacology**	*n*= 12	Min: second Max: third	Min: 60 h Max: 150 h
**Pharmacology II/ applied or clinical**	*n*= 9	Min: second Max: fourth	Min: 60 h Max: 240 h

### Pharmacology teachers

To identify the profile of pharmacology teachers, we carried out a curricular survey of 14 universities and detected a sample of 67 subjects. With this, we discovered that approximately 62% of pharmacology teachers were physicians, and 73% of has Ph.D. degrees as shown in Table 4.

**Table 4:** Academic profile of Pharmacology teachers

	***Total (n= 67)***
**Undergraduate**	Medical (42) Pharmacist (12) Biologist (9) Other (4)
**Graduate**	Ph.D. (49) Master (10) Other (8)

In this scenario, we interviewed 16 pharmacology teachers to understand their profiles and motivation. As shown in Table 5, most pharmacology teachers (75%) work more than 30 hours a week and more than 90% of them share responsibility for the subject with other teachers. In addition to teaching, most of these professors still divide their time orientating scientific initiation students, assisting patients and performing other related activities.

**Table 5:** Work profile of pharmacology teachers

	***Total (n= 16)***
**Workload (per week)**	< 10 h (1) 10 – 20 h (3) 20 – 30 h (0) 30 – 40 h (9) > 40 h (3)
**Number of pharmacology teachers in medical course**	1 teacher (1) 2 teachers (4) 3 teachers (3) 4 teachers (2) 5 teachers (2) > 5 teachers (4)
**Developed activities at medical school**	Only teach (1) Teach and orient scientific initiation students (6) Teach, orient scientific initiation students, and made patient care (4) Other (5)

In our survey, we identify that 81.3% of the teachers considered pharmacology to be a complex subject, among which, 18.8% of these teachers believe that it is a very complex subject and 87.5% classified pharmacology as a subject of intermediate level as shown in Table 6. All of the teachers responded that pharmacology is a very important subject. These teachers reported that pharmacology is substantially interdisciplinary (56.3%) and multidisciplinary (31.3%) as shown in Table 6. All of the teachers considered that contents from other subjects, such as mathematics, chemistry, biochemistry, and physiology, are also applied when teaching pharmacology.

**Table 6:** Teachers perception for pharmacology subject

	***Total (n=16)***
**Subject complexity**	Very complex (3) Complex (13) Little complex (0)
**Importance of the subject**	Very important (12) Important (4) Unimportant (0)
**Considered level**	Basic (2) Intermediate (14) Professional (0)
**The character of the subject**	Isolated (0) Multidisciplinary (5) Interdisciplinary (9) Other (2)
**Students’ approbation**	> 90% (5) 70 - 90% (8) 40 - 70% (3) < 40% (0)

The teachers were asked to report the percentage of students that passed the pharmacology exams. The approval rate most recurrent was 70% to 90% among the interviewed teachers as shown in Table 6.

**Fig. 2:**
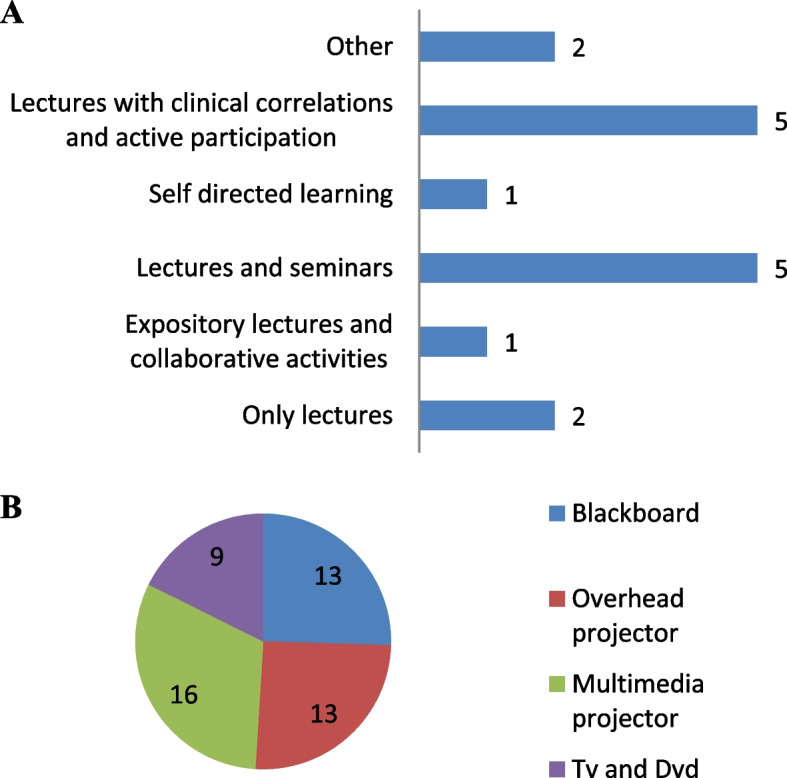
Teaching strategies used in pharmacology classes in the state of Rio de Janeiro. Teaching strategies practiced by the pharmacology teachers (**A**) and educational resources used (**B**) in the medical schools in the state of Rio de Janeiro (*n*= 16 interviewed teachers).

The use of expository lectures was the main teaching strategy used and the teachers also reported that all their students were encouraged to participate actively during the classes. The teachers also reported that they aimed to build a connection between clinical practice and the topics discussed in class. All the teaching strategies that were used by the teachers in the present study are shown in Fig. 2A. The multimedia projector was a commonly used resource, in addition to the blackboard and the overhead projector (Fig. 2B).

### Medical students

Most of the students who answered the questionnaire were women (71.9%), with a mean age of 23 years, attending the fifth year of medical school in public (68.5%) and private (31.5%) universities in Rio de Janeiro state as shown in Table 7.

**Table 7:** Gender and academic profiles of students

	***Total (n=89)***
**Sex**	Women (64) Men (25)
**Age (years)**	23.4^a^ (20 - 30)^b^
**Periods (classes)**	9^c^ (5 – 11)^b^
**University**	Public (61) Private (28)

^a^Mean

^b^Min – Max

^c^Median

Initially, we aimed to discern the students’ satisfaction and motivation level for pharmacology as a subject. A significant number (55%) of the medical students responded as being discontented and poorly motivated. Only 8 of the medical students (8.9 %) responded as completely satisfied and very motivated. The other 35.9% (32) of the students responded as motivated, as shown in Table 8.

**Table 8:** Motivation for pharmacology subject

	***Total (n= 89)***
**Motivation**	Very motivated (8) Motivated (32) Poorly motivated (49) Unmotivated (0)
**Performance**^**a**^	Excellent (4) Good (40) Satisfactory (45)
**Importance of the subject**	Very important (44) Important (44) Unimportant (1)
**Subject complexity**	Very complex (41) Complex (46) Little complex (2)
**Considered level**	Basic (8) Intermediate (54) Professional (27)

^a^Excellent = grade between 9 and 10, Good = grade between 7 and 8, and Satisfactory = grade between 6 and 7

We investigated if this low motivation is related to performance in the discipline. According to what the students reported, only 4 (4.5%) had an excellent performance (grade A or 9-10) in the subject; while 40 (45%) performed well (grade B or 7-8), and 45 (50.5%) performed satisfactorily (grade C or 6-7) in pharmacology (Table 8). The students almost unanimously recognized that this discipline is important and at the same time complex, considering its demand at an intermediate to professional level (Table 8).

Another factor that we investigated was whether the type of class taught in pharmacology could be affecting student motivation. In agreement with the teachers’ responses, 90% of the students stated that the main teaching strategy was the expository lecture, although guided exercises and seminars were also used sometimes (7.8% and 2.2%, respectively), as demonstrated in Table 9. When we asked what kind of pharmacology class they preferred, the students demonstrated a clear preference: 69.6% of the students liked expository lectures. Conversely, many students preferred more active teaching strategies. Approximately 25.8% of the students answered that the ideal teaching strategy was an introductory problem followed by an explanation and discussion concerning the main theme - Problem-Based Learning (PBL) methodology (Table 9). This item on the questionnaire allowed for an open response and several students replied with an answer that highlighted the relevance of theoretical training and exercises to increase overall test performance in their pharmacology course.

**Table 9:** Summary of pharmacology classes

	***Total (n= 89)***
**Predominant classes**	Expository (80) Seminars (2) Guided exercises (7)
**Preferred class type**	Expository (62) PBL (23) Problematized class (1) Other (3)
**Predominantly used resource**	Multimedia projector (64) Overhead projector (14) Blackboard (11)
**Were clinical examples presented?**	No (47) Yes (42) Frequently (5) Eventually (10) Rarely (27)
**Practical classes**	No (89) Yes (0)
**Assessments**	Tests (89) Projects and seminars (5) Participation in classes (1) Guided exercises (1)
**Interdisciplinary classes**	No (65) Yes (24) Frequently (1) Eventually (10) Rarely (13)
**Need for other knowledge**	No (26) Yes (63) Frequently (4) Eventually (33) Rarely (26)

Most students reported the use of a multimedia projector as the main resource (71.9%), which was followed by the overhead projector (15.7%) and blackboard (12.3%). Many students (79.7%) stated that computers and educational software were relevant to improving their learning (data not shown). When we asked why, the following main keywords were used: “helps memorize content”; “it is an environment where it is possible to perform exercises”; and “facilitation of the visualization of drugs’ mechanisms of action”.

A significant number of students stated that the teachers did not use, or rarely used, clinical examples during the classes (52.8% and 30.3%, respectively) (Table 9); while most of the teachers declared that usually clinical examples are used during the classes. Additionally, the medical students reported that there were no practical classes and that they were assessed using written tests (Table 9). In the open-response portion of this item, several students included seminars as a partial performance assessment.

Most students reported that they did not have interdisciplinary classes (73%). Some students reported that they did not need to read any non-pharmacology sources and that they did not need different skills in any other area to study pharmacology. Twenty nine point two percent of the students that were enrolled in the present study stated that they had never used different skills from other subjects to study pharmacology, and the same percentage of students stated that they rarely use other textbooks in addition to the pharmacology textbook (Table 9). The other 41.6% of the students reported that they used other subjects, such as physiology, biochemistry, among others, to better understand pharmacology’s concepts.

At the end of students’ questionnaires, there was an open-response field to comment freely. The predominant response was related to the curricular distance to the clinical subjects and the lack of integration between pharmacology and other medical practices. Some students suggested changing the pharmacology course for a time further in the medical program when students are taking a medical clinical internship.

## Discussion

Medical education attracts worldwide interest due to its potential social impact on health and educational institutions as well as the health care services provided to society. In the context of medical education pharmacology education can be considered a key point, since physicians are responsible for prescribing in most countries [36, 37]. The ability to prescribe safely and effectively in common clinical situations is an essential skill for recent medical graduates [38].

Medical education attracts an increasing number of groups that debate the definition of public policies and general issues related to health and education. According to an estimation by The World Federation for Medical Education, there were 2,900 medical schools in the world in 2017 [39]. This number has remained stable in developed countries. However, in Brazil, there are 361 medical schools for a total population of approximately 216 million [1]. This number of medical schools is higher than in China (158) and the United States (193, which 155 are allopathic (MD) and 38 are osteopathic (DO)), which have approximately 1.4 billion and 331 million of the population, respectively [40-42].

Brazilian education is ruled by a federal law from 1996 named "Law of Guidelines and Bases". This law establishes that higher education must promote teaching, and also encourage the development of scientific research and extension projects. However, a strong possibility exists that some medical schools do not perform research activities regularly [43]. We believe that the teachers’ dedication to academic activities at the university allowed them to teach, develop research and extension projects, and participate in clinical care at the university hospital, which makes a difference in the student’s education. Future prospective studies should be conducted to investigate ways to improve medical education. Additionally, teachers from private medical schools are often part-time teachers that teach at more than one school, which results in a heavy workload from different universities and different courses, such as Nursing, Pharmacy, and others. This heavy workload may contribute to poor quality of education for the students when compared to that provided by teachers that are exclusively dedicated to one school and one course.

The overall curricula was similar to the medical schools in our study. The average number of hours that were dedicated to pharmacology subjects was 144, with minor variations between the courses analyzed. This number was similar to or slightly greater than what has been observed in other countries, such as the United States, United Kingdom, Canada, and Mexico [44-46]. Conversely, pharmacology courses are usually taught in one or two parts, and only half of these courses attempt to address clinical pharmacology. The above-mentioned countries have offered basic and clinical pharmacology as subjects for medical students.

In the United States, the United Kingdom, the Netherlands, and the Canada, clinical pharmacologist profession exists (physician or non-physician) and many authors have reported problems related to clinical pharmacology education [6, 33, 45]. In the present study, clinical pharmacology appeared to be a more critical course in pharmacology education. More than 50% of the medical students stated that they received no clinical examples during the pharmacology classes and approximately 30% of the students reported that examples were rare; however, the teachers reported that clinical pharmacology was often addressed in classes. This apparent discrepancy between the responses of the students and the teachers is a complex phenomenon. Many factors are involved in the students’ perceptions of the in-class teachers’ practices, including the students’ ratings of the teachers [47]. Students’ ratings of their teacher performance are widely used in higher education institutions to evaluate teaching effectiveness and education quality. Using these rating scales, students rate their teachers’ knowledge of the subject matter, enthusiasm, organization, and presentation of the lesson. It is argued that student evaluation rating scales should also address students’ evaluation of their own learning experiences in addition to the facilitation role of the teacher [48].

These potential problems in clinical pharmacology education could be related to the prescribers’ errors and several adverse events [49]. For instance, a British study showed that junior doctors feel poorly prepared by their training in clinical pharmacology and commonly make prescribing errors [33]. A recent study with twenty-seven countries of the European Union indicates that this pattern remains similar in pharmacology and clinical pharmacology [50]. They found that 8% of medical students in their final year felt "not prepared" to prescribe and 61% felt "fairly prepared". Several authors have stated that an improvement in clinical pharmacology may be related to the shift in the medical curricula away from discipline-based to more integrated, problem-based programs, eliminating the need for formal courses and assessments in clinical pharmacology and therapeutics [6]. Unfortunately, teaching pharmacological basic concepts is an arduous process and many students fail to relate these concepts to practice and clinical experience [51]. However, in at least half of Brazilians medical schools traditional curricula are used and no clinical pharmacology course is given. Interdisciplinary approaches and the use of active methodologies are still the least popular in classes in Brazilian medical schools. Our data revealed a lack of integration between the subjects, including those in the basic and clinical curricula. Integrative approaches to the presentation of curricular materials in undergraduate medical education have received considerable attention because they present a cohesive approach to medical problems and are believed to increase students’ motivation [46, 52, 53]. Considerable interest has been expressed in a pharmacology education that is capable of promoting the acquisition of integrated basic and clinical scientific knowledge and contributing to the development of clinical reasoning skills.

Changes in pharmacology education are being driven by various forces. The exponential increase in biochemical and molecular knowledge, emergent pathologies and the diminished efficacy of a significant number of drugs may be a few of these forces. The results of the present study revealed few differences in the learning approaches. The expository lecture was reported to be the main approach that was used to teach pharmacology. Variations such as seminar presentations and clinical correlations, which were used during classes, were also reported by the pharmacology teachers. Moreover, there were significant differences between the teachers’ responses and the student's responses. Students reported that expository lectures were the only teaching strategy used. Currently, several alternative strategies for teaching pharmacology are available, including problem-based learning, simulated practices, peer assessment, interactive computer-based learning, virtual learning environments, and integrative and collaborative medical courses. Interestingly the main teaching approach was the expository lecture, which is a passive strategy for teaching. This approach agreed with the student's preferences. Alternatively the responses concerning the motivational and class preference aspects were conflicting. If the teacher’s strategies are the same as those preferred by the medical students, why are the students not motivated and satisfied? Teaching strategies may be most effective when the learners can think and talk together, discuss ideas, and analyze and solve problems. The motivation scale is a useful parameter that avoids constant teacher mediation. This result is intriguing because in the present study the students preferred the expository lecture and they were not content during their classes. Much discussion has been made regarding active methodologies and the difficulty in implementing them by teachers and schools; however, there has been little discussion concerning the student’s barriers to implementing these active methodologies. In general, these active methodologies force students to work hard during classes and in other extra-class situations. These conflicting results should be investigated in future studies.

The teachers observed that many students, after only a few lectures, failed to comprehend much of the lecture material. These didactic lectures were presented via PowerPoint software and were distributed as black-and-white handouts. Many students appeared to focus too much on the details instead of on the central idea of the lectures. The level of noise in the classroom grew as the students became lost and became increasingly inattentive during the lectures. These distractions negatively affected the course and class attendance declined by as much as 25% [54].

Several pedagogical strategies could be used to improve the overall students’ performances, including games, collaborative practices, and educational software, among others [54, 55]. The primary rule of an educational game is to increase students’ motivation and engagement [56]. In this context, only two teachers from two different medical schools reported using educational software. In addition, none of the students reported the use of educational software.

All respondent teachers reported that the multimedia projector was the main resource used during pharmacology classes. The multimedia projector was designed to be used for multimedia presentations. This tool allows for teaching in more than one form and combines the use of text, audio, graphics, full-motion video, show animations, simulations, and detailed images, among others. This important tool has also been used in the medical schools in the state of Rio de Janeiro exclusively as a high-definition overhead or slide projector to present static images. In a previous study a meta-analysis of 26 primary studies, which yielded 76 pair-wise comparisons of the dynamic and static visualizations, revealed a medium-sized, overall advantage of instructional animations over static pictures [57]. In this context, the current pharmacology education practices in medical schools in Rio de Janeiro are in opposition to the contemporary theories of cognitive load and multimedia learning, resulting in negative practical implications for instructional design.

The activities that take place in the laboratory are rarely used while teaching pharmacology. More than half of the medical schools have not provided these classes to their students during their pharmacology courses, which indicates that laboratory animals are not used. This finding is relevant because in Brazil and other countries ethical considerations pose several restrictions to the use of animals. In addition other factors may be related to the absence of laboratory practices, including low levels of teacher motivation and an absence of general support to perform practical classes. Even so, there is no trend to replace these laboratory pharmacological practices with animals at schools that do not offer these activities. For instance several reports show that there are learning advantages to simulation practices and other computer-based approaches when these practices are not possible [57-60].

Faculty members, especially medical ones, are often considered to be specialists in their discipline and knowledge field. This level of expertise is essential for admission to the medical school staff. However, not all professors at medical schools have the basic pedagogical knowledge to provide effective teaching and facilitate learning in the classroom, as was observed in the pharmacology departments of the medical schools in the state of Rio de Janeiro. Medical professors are extremely well-trained in their respective research fields but have insufficient or no formal teacher training [61]. A recent meeting of education for safe prescribing from 12 European countries has not discussed the question of pedagogical knowledge to teach clinical pharmacology and therapeutics [36]. This lack of training could be an important cause for the poor motivational status of medical students. Presently, graduate courses have included training in pedagogical approaches at master's and doctoral levels to better prepare future teachers. Graduate students now have to complete teaching activities as a requisite for completing their courses. This strategy to increase the teaching ability of faculty members began when it was observed a significant growth in the number of students in the graduate courses. The data show that no improvement was achieved in the pedagogical competencies of the pharmacology educators in the medical schools in the state of Rio de Janeiro.

When we compare our findings with other countries in the world we realize common problems. A great number of universities in the world have incorporated PBL for teaching pharmacology as a solution for pharmacology education [36]. However, there is no clear evidence that this change is without risk [62]. As suggested by Hudec et al., we believe that a hybrid process with PBL could improve the teaching-learning process. In addition, clinical pharmacology should have been implemented last year, but with an interdisciplinary approach. In the short term, medical students would likely have to learn a new field of pharmacology, named systems pharmacology [63].

More active pedagogical strategies and the use of technological resources need to be implemented to adequately prepare students for medical demands at a global level. Public institutions are maintained by the federal or state governments in Brazil and these courses are offered to the students free of charge, while the privately funded (profit or non-profit) institutions charge for their services, but they also offer scholarships to some students.

### Study limitations

In the teachers’ questionnaires we asked about the approval rate without considering the grades, since they stated that most students passed the exams. Thus, it is plausible to have a high percentage of approved students, as reported by the teachers, even with low grades, as shown in the student’s responses to the questionnaires.

It should be noted that these resources are consistent with expository lectures and that resources such as digital platforms were not mentioned, since the research took place before the COVID-19 pandemic. In the pandemic scenario they became one of the main, if not the main, educational resource. This trend is at odds with the teachers’ concerns that were mentioned above, in terms of integration of basic concepts and clinical practice. In addition, considering that all participants are volunteers a bias may be present because they are more concerned with the need to learn pharmacology and make good prescriptions.

## Conclusions

The study pointed to a growing number of private universities in the state of Rio de Janeiro which are mostly located in the metropolitan region of the state. It also suggests that traditional passive learning was the main way of learning pharmacology in most medical schools in Rio de Janeiro before the COVID-19 pandemic. In general, pharmacology teachers present a high workload and perform other academic and clinical-related activities. Finally, medical students are poorly motivated which may be related to their performance in assessments due to the complexity of the subject otherwise to passive pedagogical used strategies. We suggest that the teaching activities should be more concatenated with the consolidated practices that can help future doctors to carry out their activities more safely, including the practice of prescribing medication. These contributions, besides improving the teaching of pharmacology, can help to minimize medication errors.

## Supplementary Information

**Additional file 1.** Teachers’ questionnaire in English.

**Additional file 2.** Teachers’ questionnaire in Portuguese.

**Additional file 3.** Students’ questionnaire in English.

**Additional file 4.** Students’ questionnaire in Portuguese.

**Additional file 5.** Survey data.

## Data Availability

All data generated or analyzed during this study are included in this published article and its supplementary information file.
