# Supplementary material for: Teaching and learning pharmacology in Brazil before COVID-19 pandemic: a case study in Rio de Janeiro
Source: BMC Med Educ. 2023 Jun 23;23:471. doi: 10.1186/s12909-023-04437-4 (PMC10288696; doi:10.1186/s12909-023-04437-4)
Supplement: Supplementary file 1 — Additional file 1. Teachers’ questionnaire in English. [file 12909_2023_4437_MOESM1_ESM.pdf]

Pharmacology Teaching

1. TERMS OF FREE AND INFORMED CONSENT

(According to Resolution No. 196, of the National Health Council of October 10, 1996)

* **1. You are being invited to participate in the research “teaching pharmacology in medical schools in the state of rio de janeiro”. You were selected for your involvement as a professor or responsible for the discipline and/or department of pharmacology of the medical course and your participation is voluntary. At any time, you can withdraw your participation and withdraw your consent. Your refusal will not harm your relationship with the researcher, with the coordination and other professors of your medical course or with your HEI.**

The investigated problem: The main objective of this study is to know the practices currently used in the teaching of pharmacology in medical schools in the State. In a second step, you will be invited to receive and use software as a pedagogical tool in the pharmacology course. With this we intend to know the advantages and disadvantages of this tool, as well as confront it with practices and other traditional tools already used.

Procedure: Your participation in this research will consist of answering a structured questionnaire, with information related to the pharmacology discipline, as well as the practices developed by the professors of this discipline in this HEI. Risks: There are no risks associated with your participation. Benefits: By accepting to participate in this research, you will be contributing to a more detailed knowledge of the practices that are developed for the teaching of pharmacology in medical schools in the State and also contributing to its improvement. In addition, later, you will be receiving a didactic tool (software) that may be useful in the teaching and learning relationships of your subject, further improving student and teacher performance. Confidentiality: The information obtained through this survey will be confidential and we ensure the secrecy of your participation. The data will not be disclosed so that your identification is possible. We do not intend to compare medical schools, but rather “map” the teaching of pharmacology in our state. Under no circumstances will your HEI be identified. The results will be disseminated in presentations or publications with scientific and educational purposes. Cost and payment: Participating in this survey will incur no cost to you or your institution. As a volunteer, you will also not receive any cash value for participating. We do not intend to compare medical schools, but rather “map” the teaching of pharmacology in our state. Under no circumstances will your HEI be identified. The results will be disseminated in presentations or publications with scientific and educational purposes. Cost and payment: Participating in this survey will incur no cost to you or your institution. As a volunteer, you will also not receive any cash value for participating. We do not intend to compare medical schools, but rather “map” the teaching of pharmacology in our state. Under no circumstances will your HEI be identified. The results will be disseminated in presentations or publications with scientific and educational purposes. Cost and payment: Participating in this survey will incur no cost to you or your institution. As a volunteer, you will also not receive any cash value for participating.

As it is an ONLINE questionnaire, its acceptance will be conditioned to clicking on the respective answer box. Upon acceptance, you will receive a copy of this term, so be sure to fill in the e-mail field in the questionnaire. In this term, you will also demonstrate your interest in participating in the second phase of this research, related to the use of the software. To do so, click on the corresponding box.

of response, also in this term. The responsible researchers are at your disposal for any clarification on this term, as well as other information relevant to this research.

Researchers: Antonio A Fidalgo-Neto, Renato M Lopes and Luiz A Alves. Lab. of Cellular Communication, IOC/FIOCRUZ and Prog. of Post-Grad. in teaching Biosciences. Phones: (21) 25601287 r. 173. Lab. of Educ. Professional in Laboratory Techniques in Health. EPSJV/FIOCRUZ. Emails: fidal go@ioc.fiocruz.br , renatoml@fiocruz.br and alveslaa@ioc.fiocruz.br

- I declare that I understand the objectives, risks and benefits of my participation in the research and I agree to participate.
- I also wish to be contacted to participate in the second stage of the research by receiving and using the software.
- No, I do not agree to participate in the research.

2. Questionnaire

Thanks so much for participating! You will not lose more than 30 minutes to answer this questionnaire, however, your contribution will be invaluable.

2. What's your name?(optional)

* **3. Enter your email. (will only be used for sending a copy of the consent document)**

* **4. What is your academic background? (graduation)**

* **5. Inform the institution where you completed your degree.**

* **6. What is your maximum title?**

* **7. What institution did you graduate from?**

* **8. What is your total teaching hours?**

- less than 10 hours
- between 10 and 20 hours
- between 20 and 30 hours
- between 30 and 40 hours
- greater than 40 hours

9. Do you have another occupation? (If so, let me know!)

10. If you have another occupation, what is the workload?

- less than 10 hours
- between 10 and 20 hours
- between 20 and 30 hours
- between 30 and 40 hours
- greater than 40 hours

* **11. How many professors are responsible for the subject (and related subjects, eg Pharmaco I, II etc.)**

- 1
- two
- 3
- 4
- 5
- more than 5

* **12. There are: (If applicable, check more than one option)**

- Doctors
- Pharmacists
- Biomedical
- nurses
- dentists
- Others

Other (please specify)

* **13. Of the total number of pharmacology professors, mark the number of professors with the respective maximum degrees.(USE NUMBERS ONLY)**

doctors

masters

specialists

Graduation

Pharmacology Teaching

*** 14. At your medical school, pharmacology professors:**

- only teach classes
- teach classes and guide scientific initiation students
- teach classes, guide scientific initiation students and provide clinical care
- Other (please specify)

*** 15. Are there other activities developed by the subject's professors not mentioned above?**

- No
- Yes (please specify)

*** 16. Is pharmacology given in just one module, or is it broken down in some way?**

- Yes
- No (please advise)

*** 17. What is the course load (if more than one, please inform)**

*** 18. Is there a discipline that deals separately with clinical pharmacology?**

- No
- Yes
- Other (please advise)

*** 19. Considering the content contemplated in the text book “GOODMAN & GILMAN - AS BASES PHARMACOLOGICAS DA THERAPEUTICA , 11th ED. 2007 by BRUNTON, LAURENCE L.; LAZO, JOHN S. AND PARKER, KEITH L.” Is there a topic not covered in your department's program?**

- No, all topics contained in the book are covered.
- Yes, but the most important topics are covered
- Yes, they are not addressed as there is not enough time.
- Yes, not all topics in the book are relevant.
- Other (please specify)

3.

*** 20. Making a comparative analysis with other disciplines in your medical course, and considering the general performance of students, would you classify pharmacology as:**

- very complex
- complex
- within reason
- It is a discipline that demands little from the student.
- Other (please specify)

*** 21. In your perception, how do you think students, in general, see the discipline in relation to its relevance?**

- Very important
- Important
- Just like all the others in the basic cycle
- Less important
- They don't understand the importance of discipline
- Other (please advise)

Pharmacology Teaching

*** 22. In your perception, how do you think students, in general, see the discipline in relation to its degree of difficulty?**

- very complex
- complex
- within reason
- It is a discipline that demands little from the student.
- Other (please advise)

*** 23. Circle the number that represents the average pass rate of students in the pharmacology course. (Use for calculation only those who completed the course)**

- >90%
- between 70% to 90%
- between 40% and 70%
- <40%
- Other (please advise)

*** 24. Regarding the inclusion of the discipline in the medical course curriculum, would you classify pharmacology as a discipline:**

- Basic
- intermediate
- Professional
- Other (please advise)

*** 25. For you, pharmacology is:**

- an isolated science
- a multidisciplinary science
- an interdisciplinary science
- Other (please advise)

*** 26. In your opinion, what would be the best position for pharmacology within the curriculum of your medical course?**

*** 27. Do you and your colleagues use knowledge from other disciplines (such as mathematics, physics, chemistry, biochemistry and physiology) during pharmacology classes?**

- No
- yes, rarely
- yes, eventually
- yes, often
- Other (please advise)

*** 28. Students are encouraged to read scientific journals (in English)**

- No
- yes, rarely
- yes, eventually
- yes, often
- Other (please advise)

4.

*** 29. Does the HEI or especially the department make computers and Internet access available for students to use?**

- No
- Yes, not enough
- Yes, in sufficient numbers
- Other (please advise)

*** 30. Does the library provide adequate access to textbooks (in number and diversity)?**

- No
- Yes
- Other (please advise)

31. Does the library provide access to scientific journals (national and international)?

- No
- Yes, only paper-based journals
- Yes, only journals subscribed online (e.g. CAPES Portal)
- Yes, only journals signed on paper and signed online (for example, CAPES Portal)
- Other (please advise)

* **32. Does the subject program contemplate and do the professors effectively carry out practical classes?**

- No
- Yes - How many over the period?

33. If practical classes are held, do they use laboratory animals?

- No
- Yes
- Other (please advise)

* **34. Does your department use any specific software as a didactic resource for teaching pharmacology?**

- No
- Yes: Which one(s)?

35. If the previous answer was “Yes”, what is the frequency of use?

- Once per term or module
- Twice per term or module
- Three times per term or module
- all month
- Every week
- Other (please advise)

Pharmacology Teaching

*** 36. Do you, in isolation, use any specific software as a didactic resource for teaching pharmacology?**

- No
- Yes: Which one(s)?

37. If the previous answer was "Yes", what is the frequency of use?

- Once per term or module
- Twice per term or module
- Three times per term or module
- all month
- Every week
- Other (please advise)

*** 38. How are the classes usually taught by you and your department colleagues?**

- Predominantly expository
- Expository and collaborative
- In addition to exposing the content, students are encouraged to present seminars
- It is common to conduct studies
- Classes are always accompanied by practical and clinical examples. Students are encouraged to actively participate.
- Other (Please advise)

*** 39. What are the resources used? (Number in descending order of use)**

- blackboard and chalk
- White board
- overhead projector
- Multimedia projector
- Television and DVD
- Other (please advise)

*** 40. Do you and your department follow any specific pedagogical line?**

- No
- Yes, which one(s)
