# Supplementary material for: Teaching and learning pharmacology in Brazil before COVID-19 pandemic: a case study in Rio de Janeiro
Source: BMC Med Educ. 2023 Jun 23;23:471. doi: 10.1186/s12909-023-04437-4 (PMC10288696; doi:10.1186/s12909-023-04437-4)
Supplement: Supplementary file 2 — Additional file 2. Teachers’ questionnaire in Portuguese. [file 12909_2023_4437_MOESM2_ESM.pdf]

Ensino de Farmacologia

1. TERMO DE CONSENTIMENTO LIVRE E ESCLARECIDO

(Conforme a Resolução nº 196, do Conselho Nacional de Saúde de 10 de outubro de 1996)

* 1. Você está sendo convidado(a) para participar da pesquisa “ensino de farmacologia nas escolas médicas do estado do rio de janeiro”. Você foi selecionado (a) por seu envolvimento como docente ou responsável pela disciplina e/ou departamento de farmacologia do curso de medicina e sua participação é voluntária. A qualquer momento você pode desistir de participar e retirar seu consentimento. Sua recusa não trará nenhum prejuízo em sua relação com o pesquisador, com a coordenação e demais docentes do seu curso médico ou com a sua IES.

O problema investigado: O objetivo principal desse estudo é conhecer as práticas atualmente utilizadas no ensino da farmacologia das escolas médicas do Estado. Em uma segunda etapa, você estará sendo convidado a receber, e utilizar um software como ferramenta pedagógica no curso de farmacologia. Pretendemos com isso conhecer as vantagens e desvantagens desta ferramenta, bem como confrontá-la com práticas e outras ferramentas tradicionais já utilizadas.

Procedimento: Sua participação nesta pesquisa consistirá em responder a um questionário estruturado, com informações relacionadas à disciplina farmacologia, bem como às práticas desenvolvidas pelos docentes desta disciplina nesta IES.

Riscos: Não existem quaisquer riscos relacionados com a sua participação.

Benefícios: Ao aceitar participar desta pesquisa você estará contribuindo para o conhecimento mais detalhado das práticas que são desenvolvidas para o ensino da farmacologia nas escolas médicas do Estado e contribuindo também, para a sua melhoria. Além disso, posteriormente, você estará recebendo uma ferramenta didática (software) que poderá ser útil nas relações ensino e aprendizagem da sua disciplina, melhorando ainda mais o desempenho discente e docente.

Confidencialidade: As informações obtidas através desta pesquisa serão confidenciais e asseguramos o sigilo sobre a sua participação. Os dados não serão divulgados de modo que seja possível a sua identificação. Não tempos a intenção de comparar as escolas médicas e sim “mapear” o ensino da farmacologia em nosso estado. Em nenhuma hipótese a sua IES será identificada. Os resultados serão divulgados em apresentações ou publicações com fins científicos e educativos.

Custo e pagamento: Participar dessa pesquisa não implicará em nenhum custo para você ou para a sua instituição. Como voluntário, você também não receberá qualquer valor em dinheiro pela participação.

Por se tratar de um questionário ONLINE, o seu aceite estará condicionado ao clique na respectiva caixa de resposta. Ao aceitar, você receberá uma cópia deste termo, por isso não deixe de preencher no questionário, o campo correio eletrônico. Neste termo, você também demonstrará o seu interesse em participar da segunda fase desta pesquisa, relacionado ao uso do software. Para isso clique na respectiva caixa

de resposta, também neste termo. Os pesquisadores responsáveis estão a inteira disposição para quaisquer esclarecimentos sobre este termo, bem como outras informações pertinentes a esta pesquisa.

Pesquisadores: Antonio A Fidalgo-Neto, Renato M Lopes e Luiz A Alves.
Lab. de Comunicação Celular, IOC/FIOCRUZ e Prog. de Pós-Grad. em ensino de Biociências. Telefones: (21) 25601287 r. 173. Lab. de Educ. Profissional em Técnicas Laboratoriais em Saúde. EPSJV/FIOCRUZ. Emails: fidalgo@ioc.fiocruz.br, renatoml@fiocruz.br e alveslaa@ioc.fiocruz.br

- Declaro que entendi os objetivos, riscos e benefícios da minha participação na pesquisa e aceito participar.
- Desejo também, ser contatado para participar da segunda etapa da pesquisa recebendo e utilizando o software.
- Não, não aceito participar da pesquisa.

2. Questionário

Muito obrigado por participar! Você não perderá mais que 30 minutos para responder este questionário, contudo, a sua contribuição será inestimável.

2. Qual o seu nome?(opcional)

____

* 3. Digite o seu e-mail. (será usado apenas para o envio da cópia do documento de consentimento)

* 4. Qual a sua formação acadêmica? (graduação)

* 5. Informe a instituição onde concluiu a sua graduação.

* 6. Qual a sua titulação máxima?

* 7. Qual a instituição onde você concluiu?

* 8. Qual a sua carga horária total docente?

- menor que 10 horas
- entre 10 e 20 horas
- entre 20 e 30 horas
- entre 30 e 40 horas
- maior que 40 horas

9. Você tem outra ocupação? (caso tenha, informe!)

10. Caso você tenha outra ocupação, qual é a carga horária?

- menor que 10 horas
- entre 10 e 20 horas
- entre 20 e 30 horas
- entre 30 e 40 horas
- maior que 40 horas

* 11. Quantos professores são responsáveis pela disciplina (e disciplinas relacionadas, ex. Farmaco I, II etc.)

- 1
- 2
- 3
- 4
- 5
- mais de 5

* 12. No quadro de professores responsáveis pela disciplina existem: (Se for o caso, marque mais de uma opção)

- Médicos
- Farmacêuticos
- Biomédicos
- Enfermeiros
- Odontólogos
- Outros
- Outro (por favor especifique)

* 13. Do quadro total de professores de farmacologia, assinale o número de professores com as respectivas titulações máximas.(UTILIZE SOMENTE NÚMEROS)

Doutores

Mestres

Especialistas

Graduação

Ensino de Farmacologia

* 14. Na sua escola médica, os professores de farmacologia:

- somente ministram aulas
- ministram aulas e orientam alunos de iniciação científica
- ministram aulas, orientam alunos de iniciação científica e fazem atendimento clínico
- Outro (por favor, especifique)

* 15. Existem outras atividades desenvolvidas pelos docentes da disciplina não mencionadas acima?

- Não
- Sim (por favor, especifique)

* 16. A farmacologia é dada em apenas um módulo, ou ela é de alguma forma desmembrada?

- Sim
- Não (por favor, informe)

* 17. Qual a carga horária da disciplina (se for mais de uma, por favor informe)

* 18. Existe uma disciplina que trata isoladamente da farmacologia clínica?

- Não
- Sim
- Outro (por favor, informe)

* 19. Considerando o conteúdo contemplado no livro Texto “GOODMAN & GILMAN - AS BASES FARMACOLÓGICAS DA TERAPÊUTICA , 11ª ED. 2007 de BRUNTON, LAURENCE L.; LAZO, JOHN S. E PARKER, KEITH L.” existe algum tópico não contemplado no programa do seu departamento?

- Não, todos os tópicos contidos no livro são abordados.
- Sim, porém os tópicos mais importantes são abordados
- Sim, não são abordados pois não há tempo suficiente.
- Sim, nem todos os tópicos do livro são relevantes.
- Outro (por favor, especifique)

3.

* 20. Fazendo uma análise comparativa com outras disciplinas do seu curso médico, e considerando o desempenho geral dos estudantes, você classificaria a farmacologia como:

- Muito complexa
- Complexa
- Dentro do razoável
- É uma disciplina que exige pouco do aluno.
- Outro (por favor, especifique)

* 21. Na sua percepção, como você acha que os alunos, de modo geral, veem a disciplina em relação à sua relevância?

- Muito importante
- Importante
- Assim como todas as outras do ciclo básico
- Menos importante
- Não entendem a importância da disciplina
- Outro (por favor, informe)

Ensino de Farmacologia

* 22. Na sua percepção, como você acha que os alunos, de modo geral, vêem a disciplina em relação ao seu grau de dificuldade?

- Muito complexa
- Complexa
- Dentro do razoável
- É uma disciplina que exige pouco do aluno.
- Outro (por favor, informe)

* 23. Assinale o número que representa a taxa de aprovação média dos alunos no curso de farmacologia. (utilize para cálculo somente aqueles que concluíram a disciplina)

- > 90%
- entre 70% a 90%
- entre 40% e 70%
- < 40%
- Outro (por favor, informe)

* 24. Em relação ao enquadramento da disciplina na grade do curso médico você classificaria a farmacologia como uma disciplina:

- Básica
- Intermediária
- Profissional
- Outro (por favor, informe)

* 25. Para você a farmacologia é:

- uma ciência isolada
- uma ciência multidisciplinar
- uma ciência interdisciplinar
- Outro (por favor, informe)

* 26. Na sua concepção, qual seria a melhor posição da farmacologia dentro da grade curricular do seu curso médico?

Ensino de Farmacologia

* 27. Você e seus colegas utilizam conhecimentos de outras disciplinas (como por exemplo, matemática, física, química, bioquímica e fisiologia) durante as aulas de farmacologia?

- Não
- Sim, raramente
- Sim, eventualmente
- Sim, Frequentemente
- Outro (por favor, informe)

* 28. Os alunos são encorajados à leitura de periódicos científicos (em língua inglesa)

- Não
- Sim, raramente
- Sim, eventualmente
- Sim, Frequentemente
- Outro (por favor, informe)

4.

* 29. A IES ou especialmente o departamento disponibiliza computadores e acesso à Internet para uso dos alunos?

- Não
- Sim, em número insuficiente
- Sim, em número suficiente
- Outro (por favor, informe)

* 30. A biblioteca oferece acesso adequado aos livros-texto (em número e diversidade)?

- Não
- Sim
- Outro (por favor, informe)

Ensino de Farmacologia

31. A biblioteca disponibiliza acesso a periódicos científicos (nacionais e internacionais)?

- Não
- Sim, somente periódicos assinados em papel
- Sim, somente periódicos assinados online (por exemplo, Portal CAPES)
- Sim, somente periódicos assinados em papel e assinados online (por exemplo, Portal CAPES)
- Outro (por favor, informe)

* 32. O programa da disciplina contempla e os professores efetivamente realizam aulas práticas?

- Não
- Sim - Quantas ao longo do período?

33. Caso sejam realizadas aulas práticas, estas utilizam animais de laboratório?

- Não
- Sim
- Outro (por favor, informe)

* 34. O seu departamento utiliza algum software específico como recurso didático para o ensino da farmacologia?

- Não
- Sim: Qual(is)?

35. Caso a resposta anterior tenha sido “Sim”, qual a frequência de uso?

- Uma vez por período ou módulo
- Duas vezes por período ou módulo
- Três vezes por período ou módulo
- Todo o mês
- Toda a semana
- Outro (por favor, informe)

Ensino de Farmacologia

* 36. Você, de forma isolada utiliza algum software específico como recurso didático para o ensino da farmacologia?

- Não
- Sim: Qual(is)?

37. Caso a resposta anterior tenha sido "Sim", qual a frequência de uso?

- Uma vez por período ou módulo
- Duas vezes por período ou módulo
- Três vezes por período ou módulo
- Todo o mês
- Toda a semana
- Outro (por favor, informe)

* 38. Como são as aulas normalmente ministradas por você e seus colegas de departamento?

- Predominantemente expositivas
- Expositivas e colaborativas
- Além da exposição do conteúdo os alunos são encorajados a apresentarem seminários
- É comum a realização de estudos dirigidos
- As aulas são sempre acompanhadas de exemplos práticos e clínicos. Os alunos são encorajados a participarem ativamente.
- Outro (Por favor, informe)

* 39. Quais são os recursos utilizados? (Numere em ordem decrescente de uso)

Quadro e giz

Quadro branco

Retroprojetor

Projetor multimídia

Televisão e DVD

Outro (por favor, informe)

* 40. Você e seu departamento seguem alguma linha pedagógica específica?

- Não
- Sim, Qual(is)
