# Supplementary material for: Teaching and learning pharmacology in Brazil before COVID-19 pandemic: a case study in Rio de Janeiro
Source: BMC Med Educ. 2023 Jun 23;23:471. doi: 10.1186/s12909-023-04437-4 (PMC10288696; doi:10.1186/s12909-023-04437-4)
Supplement: Supplementary file 3 — Additional file 3. Students’ questionnaire in English. [file 12909_2023_4437_MOESM3_ESM.pdf]

Laboratory of Cellular Communication – IOC/ FIOCRUZ

Questionnaire on the teaching of pharmacology (it is not necessary to identify yourself)

To participate, you must have already completed the Pharmacology subject(s).

Gender: ( ) Male ( ) Female Age: _________ Period: _________

1 - What was the degree of motivation you had for the discipline of Pharmacology?

( ) unmotivated
( ) highly motivated
( ) little motivated
( ) motivated

2 - In general, how were the classes taught by your pharmacology professors? (check the two most used options)

( ) Predominantly expository.
( ) Based on the presentation of seminars by the students.
( ) From the use of directed studies.
( ) Otherwise (Please advise): ________________________________

3 - Were pharmacology classes developed from practical and clinical examples?

( ) Yes (a little, reasonably or a lot) (_________________)
(   ) No

4 - What were the two main resources used in pharmacology classes? (write 1 for the most used and 2 for the second most used)

( ) Blackboard (or whiteboard) and chalk
( ) Overhead projector
( ) Multimedia projector (“data show”)
( ) Television and DVD
( ) Other (please inform) ____

5 - An “interdisciplinary” class integrates knowledge from different “disciplines”. Was it common to develop interdisciplinary classes in the discipline of Pharmacology?

( ) No
( ) Yes - if so, indicate the frequency of these classes
( ) Often

( ) Sometimes
( ) Rarely

6 - Regarding the development of a class, do you prefer:

( ) That the teacher gives the class explaining the subject and that you have the responsibility of paying attention to what is exposed by the teacher
( ) That the teacher starts the class by posing a problem or task for you to think about and that he does not explain anything that day about the problem or task presented to you
( ) The teacher should start the class by posing a problem or task for you to reflect on and only then, if necessary, explain what is needed
( ) Other way (please inform)

7- Does your institution provide computers and easy access to the Internet?

( ) No
( ) Yes, not enough
( ) Yes, in sufficient numbers

8 -Does the library provide adequate access to textbooks (in number and diversity)?

( ) No
( ) Yes

9 - What is the main textbook adopted by the discipline of pharmacology?

10 - What is your favorite pharmacology book?

11 - Did you and your colleagues use knowledge from other disciplines (such as mathematics, physics, chemistry, biochemistry and physiology) during pharmacology classes?

( ) No
( ) Yes, rarely
( ) Yes, eventually
( ) Yes, Often
( ) Other (please inform)____

12 - Did the teachers use any specific software as a didactic resource for teaching pharmacology?

( )No
( )Yes: Which one(s)? ____

13 - If the previous answer was “Yes”, what is the frequency of use?

( ) Once per term or module
( ) Two or three times per term or module
( ) All month
( ) Every week

14 - Do you use any specific software as a didactic resource for teaching pharmacology?

( ) No
( )Yes: Which and how often? ____

15 - Do you believe that the use of information technology and the use of educational software can be useful tools for learning the contents and techniques of pharmacology?

( ) No
( ) Yes
Please justify your answer:

____
____

16 - Were there practical classes in the development of the discipline of Pharmacology?

( ) No
( ) Yes - How many over a period? ( )

17 - If practical classes have been held, do they use laboratory animals?

( ) No
( ) Yes - Please inform the species used: _______________________________

18 - How were you evaluated in the discipline of pharmacology?

( ) through tests and trials
( ) through presentation of seminars or development of projects
( ) for their behavior and participation in the classroom

(   ) self-evaluation
( ) Other (please describe)____

19 - What was your performance in the discipline?

( ) Excellent (9 – 10)
( ) Good (7 – 8)
( ) Sufficient (6 – 7)
( ) Insufficient (below 6)

20 - Comparing pharmacology with other disciplines in your course curriculum, how would you rate its importance?

( ) Little important
( ) Important
( ) As well as all the others in the basic cycle
( ) Very important

21 - Among the disciplines studied so far, in relation to the level of difficulty, how would you classify pharmacology?

( ) Very complex (requires a lot from the student)
( ) Little complex (requires little from the student)
( ) Complex (requires the same from the student as other disciplines)

22 - Regarding the subject's inclusion in the medical course curriculum, would you classify pharmacology as a subject:

( ) Basic
( ) Intermediate
(   ) Professional

23 - Make comments, criticisms and suggestions about the teaching of Pharmacology and other disciplines of your medical training:

____
____
____
____
____
____
____
____
____
____
____
____

Thank you very much for his participation!

Main Investigators: Antonio Augusto Fidalgo-Neto, Renato Matos Lopes and Luiz Anastácio Alves.

E-mails: fidalgo@ioc.fiocruz.br, renatoml@fiocruz.br e alveslaa@ioc.fiocruz.br
