# Supplementary material for: Teaching and learning pharmacology in Brazil before COVID-19 pandemic: a case study in Rio de Janeiro
Source: BMC Med Educ. 2023 Jun 23;23:471. doi: 10.1186/s12909-023-04437-4 (PMC10288696; doi:10.1186/s12909-023-04437-4)
Supplement: Supplementary file 4 — Additional file 4. Students’ questionnaire in Portuguese. [file 12909_2023_4437_MOESM4_ESM.pdf]

Laboratório de Comunicação Celular FIOCRUZ / IOC
Questionário sobre o ensino de farmacologia (não é necessário se identificar).

Para participar é preciso que você já tenha concluído a(s) disciplina(s) de Farmacologia.

Sexo: ( ) Masculino   ( ) Feminino   Idade: _________ Período: _________

1 - Qual foi o grau de motivação que você teve pela disciplina de Farmacologia ?
( ) desmotivado.
( ) muito motivado.
( ) pouco motivado.
( ) motivado.

2- Em geral, como foram as aulas ministradas pelos seus professores de farmacologia? (marque as duas opções mais usadas).
( ) Predominantemente expositivas.
( ) Pautada na apresentação de seminários por parte dos alunos.
( ) A partir do uso de estudos dirigidos.
( ) De outra forma (Por favor, informe):_______________________________

3 - As aulas de farmacologia foram desenvolvidas a partir de exemplos práticos e clínicos?

( ) Sim (pouco, razoavelmente ou muito) (_________________).
( ) Não.

4 - Quais foram os dois principais recursos utilizados nas aulas de farmacologia? (escreva **1** para o mais usado e **2** para o segundo mais usado).

( ) Quadro (ou quadro branco) e giz.
( ) Retroprojetor.
( ) Projetor multimídia (“data show”).
( ) Televisão e DVD.
( ) Outro (por favor, informe) ____

5 - Uma aula “interdisciplinar” integra conhecimentos de diferentes “disciplinas”. Foi comum o desenvolvimento de aulas interdisciplinares na disciplina de Farmacologia?

( ) Não

( ) Sim - em caso afirmativo, assinale a freqüência dessas aulas.
( ) Muitas vezes   ( ) Algumas vezes   ( ) Raramente aconteceu

6 - Quanto ao desenvolvimento de uma aula você prefere:
( ) Que o professor dê a aula explicando a matéria e que você fique com a responsabilidade de prestar atenção no que é exposto pelo professor.
( ) Que o professor inicie a aula colocando um problema ou tarefa para você pensar e que não explique nada nesse dia sobre o problema ou tarefa apresentada para você.
( ) Que o professor inicie a aula colocando um problema ou tarefa para você refletir para só depois, se necessário, ele explicar o que for preciso.
( ) Outra forma (por favor, informe)

____

7- A sua Instituição disponibiliza computadores e acesso fácil à Internet?
( ) Não
( ) Sim, em número insuficiente
( ) Sim, em número suficiente

8 -A biblioteca oferece acesso adequado aos livros-texto (em número e diversidade)?
( ) Não
( ) Sim

9 - Qual o principal livro texto adotado pela disciplina de farmacologia?

____

10 - Qual o livro de farmacologia de sua preferência?

____

11 - Você e seus colegas utilizaram conhecimentos de outras disciplinas (como por exemplo, matemática, física, química, bioquímica e fisiologia) durante as aulas de farmacologia?
( ) Não
( ) Sim, raramente
( ) Sim, eventualmente
( ) Sim, Frequentemente
( ) Outro (por favor, informe)____

12 - Os professores utilizaram algum software específico, como recurso didático para o ensino da farmacologia?
( )Não
( )Sim: Qual(is)? ____

13 - Caso a resposta anterior tenha sido “Sim”, qual a frequência de uso?
( ) Uma vez por período ou módulo
( ) Duas ou três vezes por período ou módulo
( ) Todo o mês
( ) Toda a semana

14 - Você utiliza algum software específico como recurso didático para o ensino da farmacologia?
( ) Não
( )Sim: Qual e com que frequência ? ____

15 - Você acredita que o uso da informática e o uso de software educativo podem ser instrumentos úteis para a aprendizagem dos conteúdos e técnicas de farmacologia ?
( ) Não
( ) Sim
Por favor, justifique a sua resposta:

____

____

16 - Ocorreram aulas práticas no desenvolvimento da disciplina de Farmacologia?
( ) Não
( ) Sim - Quantas ao longo de um período? ( )

17 - Caso tenham sido realizadas aulas práticas, estas utilizam animais de laboratório?
( ) Não
( ) Sim - Por favor, informe as espécies usadas: __________________________________

18 - Como você foi avaliado na disciplina de farmacologia?
( ) através de provas e testes.
( ) através de apresentação de seminários ou desenvolvimento de projetos.
( ) pelo seu comportamento e participação em sala de aula.
( ) auto-avaliação.
( ) Outro (por favor, descreva)____

19 - Qual foi o seu desempenho na disciplina?
( ) Excelente (9 – 10)
( ) Bom (7 – 8)
( ) Suficiente (6 – 7)
( ) Insuficiente (abaixo de 6)

20 - Comparando a farmacologia com outras disciplinas da grade curricular do seu curso, como você classificaria a importância da mesma ?
( ) Pouco importante
( ) Importante
( ) Assim como todas as outras do ciclo básico
( ) Muito importante

21 - Dentre as disciplinas cursadas até o presente momento, em relação ao nível de dificuldade, como você classificaria a farmacologia?
( ) Muito complexa (exige muito do aluno)
( ) Pouco complexa (exige pouco do aluno)
( ) Complexa (exige do aluno o mesmo que outras disciplinas)

22 - Em relação ao enquadramento da disciplina na grade do curso médico você classificaria a farmacologia como uma disciplina:
(   ) Básica
(   ) Intermediária
(   ) Profissional

23 - Faça comentários, críticas e sugestões sobre o ensino de Farmacologia e de outras disciplinas da sua formação médica:

____

____

____

____

____

____

____

____

Muito obrigado pela sua participação!

Pesquisadores Responsáveis: Antonio A Fidalgo-Neto, Renato Matos Lopes e Luiz Anastácio Alves.

E-mails: fidalgo@ioc.fiocruz.br, renatoml@fiocruz.br e alveslaa@ioc.fiocruz.br
